# Development and Validation of a Novel LC-MS/MS Method for the Simultaneous Determination of Abemaciclib, Palbociclib, Ribociclib, Anastrozole, Letrozole, and Fulvestrant in Plasma Samples: A Prerequisite for Personalized Breast Cancer Treatment

**DOI:** 10.3390/ph15050614

**Published:** 2022-05-16

**Authors:** Lu Turković, Luka Bočkor, Oscar Ekpenyong, Tajana Silovski, Mila Lovrić, Slaven Crnković, Biljana Nigović, Miranda Sertić

**Affiliations:** 1 Department of Pharmaceutical Analysis, Faculty of Pharmacy and Biochemistry, University of Zagreb, Ante Kovacica 1, 10000 Zagreb, Croatia; lu.turkovic@pharma.unizg.hr (L.T.); bnigovic@pharma.hr (B.N.); 2 Centre for Applied Bioanthropology, Institute for Anthropological Research, Ljudevita Gaja 32, 10000 Zagreb, Croatia; lbockor@inantro.hr; 3 ADME & Discovery Toxicology, Merck & Co., Inc., South San Francisco, CA 94080, USA; oscar.ekpenyong@merck.com; 4 Department of Oncology, University Hospital Centre Zagreb, Kispaticeva 12, 10000 Zagreb, Croatia; tsilovsk@kbc-zagreb.hr; 5 Department of Laboratory Diagnostics, University Hospital Centre Zagreb, Kispaticeva 12, 10000 Zagreb, Croatia; mila.lovric@kbc-zagreb.hr; 6 Faculty of Pharmacy and Biochemistry, University of Zagreb, Ante Kovacica 1, 10000 Zagreb, Croatia; 7 Ludwig Boltzmann Institute for Lung and Vascular Research, Neue Stiftingtalstrasse 6/V, 8010 Graz, Austria; slaven.crnkovic@medunigraz.at; 8 Division of Physiology, Medical University of Graz, 8010 Graz, Austria

**Keywords:** CDK4/6 inhibitors, breast cancer, palbociclib, ribociclib, abemaciclib, anastrozole, letrozole, fulvestrant, therapeutic drug monitoring, LC-MS

## Abstract

Palbociclib, ribociclib and abemaciclib were recently approved as chemotherapeutic agents and are currently in the post-marketing surveillance phase. They are used in combination with aromatase inhibitors anastrozole and letrozole or antiestrogen fulvestrant for HR+, HER2− breast cancer treatment. Here, a novel bioanalytical LC-ESI-MS/MS method was developed for the quantitation of these six drugs in human plasma. The samples were prepared by simple protein precipitation followed by solvent evaporation. A Kinetex biphenyl column (150 × 4.6 mm, 2.6 µm) used for chromatographic analysis adequately resolved even the closely eluting aromatase inhibitors’ peaks. The mobile phase consisted of 0.1% formic acid in water and in ACN, in a linear gradient. An additional gradient step was added to eliminate the observed carry-over. The proposed method was fully validated in the relevant linear ranges covering the expected plasma concentrations of all six drugs (correlation coefficients between 0.9996 and 0.9931). The intra-day method precision (CV) ranged from 3.1% to 15%, while intra-day accuracy (%bias) was between −1.5% and 15.0%. The inter-day precision ranged from 1.6% to 14.9%, with accuracy between −14.3% and 14.6%, which is in accordance with the EMA and ICH guidelines on bioanalytical method validation. The method was successfully applied to samples from patients treated for HR+, HER2− breast cancer.

## 1. Introduction

**Figure 1:**
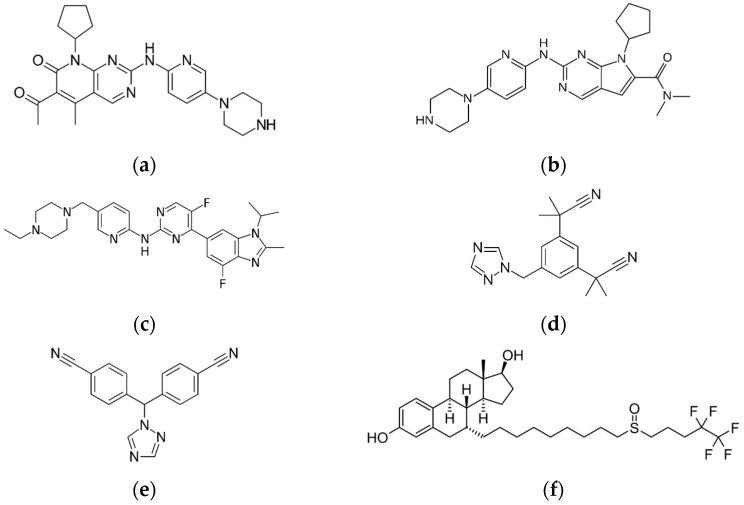
Chemical structures of (**a**) PAL, (**b**) RIB, (**c**) ABE, (**d**) ANA, (**e**) LET and (**f**) FUL.

Breast cancer is the most common type of cancer in women; it is estimated that it will affect at least one in eight women during their lifetime. It causes the highest cancer-related mortality among women globally [1]. Advanced breast cancer comprises both locally advanced breast cancer and metastatic breast cancer [2]. Although treatable, metastatic breast cancer remains virtually an incurable disease, with an overall median survival of three years [3]. Over two-thirds of breast cancer are hormone receptor-positive (HR+)/human epidermal growth factor receptor 2-negative (HER2−) tumors [4]. There has been significant advancement in endocrine therapy in the past few years, introducing combination regimens. Excellent results have been observed with palbociclib (PAL), ribociclib (RIB) and abemaciclib (ABE), which are novel anticancer agents, inhibitors of the cyclin D-dependent kinases (CDK) 4 and 6. Inhibiting the CDK4/6 proteins blocks cell division, resulting in the slowing down of cancer growth. The CDK4/6 inhibitors are considered targeted therapy, since they work differently from traditional chemotherapy, demonstrating selectivity for the specific molecules of the intracellular pathway involved in the cell division and progression. They are administered in combination with endocrine therapy—an aromatase inhibitor, anastrozole (ANA) or letrozole (LET), or with a selective estrogen receptor degrader fulvestrant (FUL) [5,6,7]. The chemical structures of these CDK4/6 inhibitors, aromatase inhibitors and FUL are shown in Figure 1. A combined approach is beneficial because endocrine therapy effectiveness is increased by the CDK4/6 inhibitors. This therapeutic combination ensures that the upstream estrogen-dependent stimulation of the cancer cell growth is neutralized, while at the same time, downstream CDK pro-proliferative effects are inhibited, consequently delaying the development of resistance to endocrine therapy [8]. 

Despite significant therapeutic benefits, CDK4/6 inhibitors still exhibit an array of serious side effects (e.g., neutropenia, thrombocytopenia, diarrhea, QTc prolongation, hepatobiliary toxicity) and a potential for inter-individual variabilities. The majority of their toxicity stems from their specific mechanism of action rather than off-target effects [8,9,10,11]. As new drugs, marketed by accelerated approval, they were under additional monitoring. The therapeutic outcomes in cancer treatment are not immediately apprehensible and bear a risk of toxicity at higher doses. Therapeutic drug monitoring (TDM) has shown to improve clinical outcomes and reduce toxicity for many anticancer drugs. Given the fact that most anticancer drugs are characterized by a steep dose–response relationship, a narrow therapeutic window and inter-individual pharmacokinetic variability, they are ideal candidates for TDM [12,13,14]. Patient age, comorbidities and possible polypragmasia, alternative and complementary therapy treatments add challenges to patient’s adherence and compliance. This can lead to suboptimal therapy outcomes, altered drug pharmacokinetics, possible side effects and drug–drug interactions. 

Therefore, it can be beneficial to accurately measure the exposure levels in these patients and tailor their doses accordingly. To this end, a sensitive and selective bioanalytical method, capable of determining very low concentrations of the analytes in patient samples, needs to be developed. 

A few liquid chromatography (LC) methods coupled to UV detection have been published for the analysis of PAL and LET [15,16] focusing on drug formulation or rat plasma samples. Several LC methods coupled to mass spectrometry have been published for the analysis of PAL, RIB and LET [17,18] or only the CDK4/6 inhibitors [19]. Methods for the analysis of ANA and FUL in combination with the CDK4/6 inhibitors are scarce. Sato et al. [13] recently published an LC-MS method for the quantitation of these six drugs with appropriate linear ranges but without full chromatographic separation of the analytes, limiting this method only to MS detection. Furthermore, high-cost, limited access stable isotope-labeled internal standards were used, which are hardly affordable for routine clinical laboratories that perform TDM for many different drugs on a daily basis. Other published methods have focused only on one of the drugs of interest, such as PAL [20,21] and RIB [22]. 

The aim of this work was to develop a simple sample preparation procedure, ensure full chromatographic separation and optimize appropriate detection parameters for the simultaneous analysis of the breast cancer drugs ABE, PAL, RIB, ANA, LET and FUL. The overall goal of our work was to apply this analytical procedure to patient plasma samples in order to gain insight into pharmacokinetic profiles in a real-life setting, as well as for therapeutic drug monitoring of these drugs to support clinicians’ dosing decisions.

## 2. Results

### 2.1. Method Development

#### 2.1.1. Optimization of the Sample Preparation Procedure

Due to the complexity of the biological matrix, adequate measures need to be taken to eliminate most interferences, enhance method selectivity and sensitivity and prolong instrument lifetime.

Acetonitrile (ACN) and methanol (MeOH) were evaluated as protein precipitation solvents at four and ten times the volume of the plasma sample, respectively. ACN was eventually chosen due to its high precipitation efficiency at lower volumes, as well as the high extraction recoveries of all the analytes (over 85%). Protein precipitation was followed by an evaporation step to minimize sample dilution, after which the dry residue was reconstituted in 65% MeOH to ensure proper solubility of all the analytes while maintaining adequate chromatographic peak shapes. 

#### 2.1.2. Optimization of the Chromatographic Conditions

In the preliminary studies, several chromatographic column chemistries were tested on an Agilent 1100 LC system equipped with a diode array (DAD) and fluorescence detector (FLD): XBridge C18 (150 × 4.6 mm, 3.5 µm, Waters), XBridge Phenyl (150 × 4.6 mm, 3.5 µm, Waters) and Kinetex Biphenyl (150 × 4.6 mm, 2.6 µm, Phenomenex). Mobile phases consisting of ultrapure water with different pH modifiers as aqueous phase, and ACN or MeOH as organic phase were tested. pH was adjusted using 0.1% formic acid pH 2.8, 0.1% acetic acid pH 3.8, 10 mM ammonium acetate buffer pH 5.3, or 10 mM ammonium bicarbonate pH 7.6. Lower pH values contributed to better peak shapes and faster elution of the basic CDK4/6 inhibitors (pKa values ranging from 3 to 8.9), with less impact on the aromatase inhibitors (pKa ≈ 2) and FUL (pKa ≈ 10) [23]. 

Favorable resolution between the closely eluting aromatase inhibitors’ peaks on the C18 and phenyl columns was achieved with MeOH in the mobile phase; however, the analysis time was extended due to strong retention of the lipophilic FUL. The biphenyl column ensured proper resolution between the peaks even with ACN as mobile phase, which enabled faster elution of FUL and thus shorter analysis time. 

The optimized conditions encompassed a biphenyl column (150 × 4.6 mm, 2.6 µm) with a mobile phase consisting of ultrapure water with 0.1% formic acid (mobile phase A), and ACN with 0.1% formic acid (mobile phase B). A linear gradient elution from 15% to 100% phase B in 8.5 min was applied. The column temperature was kept at 25 °C, while the autosampler was thermostated at 10 °C. With a flow rate of 0.4 mL/min to enable splitless LC-MS analysis, all analytes eluted within 10 min.

These conditions were transferred to an Agilent 1290 UHPLC system coupled to a QTOF mass spectrometer, where the method development procedure was finalized and optimized for use on real biological samples through careful selection of the appropriate MS parameters. The mobile phase gradient was adjusted by introducing an additional washing step to decrease the observed carry-over. 

#### 2.1.3. Optimization of the Detection Conditions

DAD and FLD were used in the preliminary studies. The chromatograms were recorded at the corresponding absorption maxima for each analyte: 360 nm for PAL, 270 nm for RIB, 320 nm for ABE and 240 nm for LET. ANA and FUL were detected by FLD at the optimized excitation wavelength of 210 nm and emission wavelength of 310 nm. All obtained LODs and the corresponding plasma levels found in literature are presented in Table 1. Due to the expected plasma concentrations being significantly lower than the LODs obtained with DAD and FLD, MS detection proved inevitable. 

First, the total ion chromatograms were scanned for the known MH^+^ masses of all the analytes, and the relative retention times of the obtained peaks were compared to those established in the preliminary method development. Thus, molecular ions were found for RIB (*m*/*z* 435.3), ABE (*m*/*z* 507.3), PAL (*m*/*z* 448.2), ANA (*m*/*z* 294.2) and FUL (*m*/*z* 607.4). However, the LET molecular ion MH^+^ *m*/*z* 286.1 showed low stability at the initial sheath gas temperature of 350 °C, breaking down extensively to a fragment *m*/*z* 217.1. Lowering the sheath gas temperature led to a significant increase in ion *m*/*z* 286.1 abundance, although the peaks of ABE and FUL decreased under these conditions, probably due to lower ionization efficiency. The temperature of 320 °C was eventually chosen, as it provided the best achievable balance in sensitivity for all the analytes. The ESI source and nozzle voltages of 4000 and 1000 V, respectively, with the TOF fragmentor voltage of 150 V, yielded the best peak shapes and sensitivities for all the analytes. Mass acquisition rates were set to three spectra per second both for the quadrupole and the TOF analyzer, ensuring the necessary sensitivity with robust peak shapes. 

**Figure 2:**
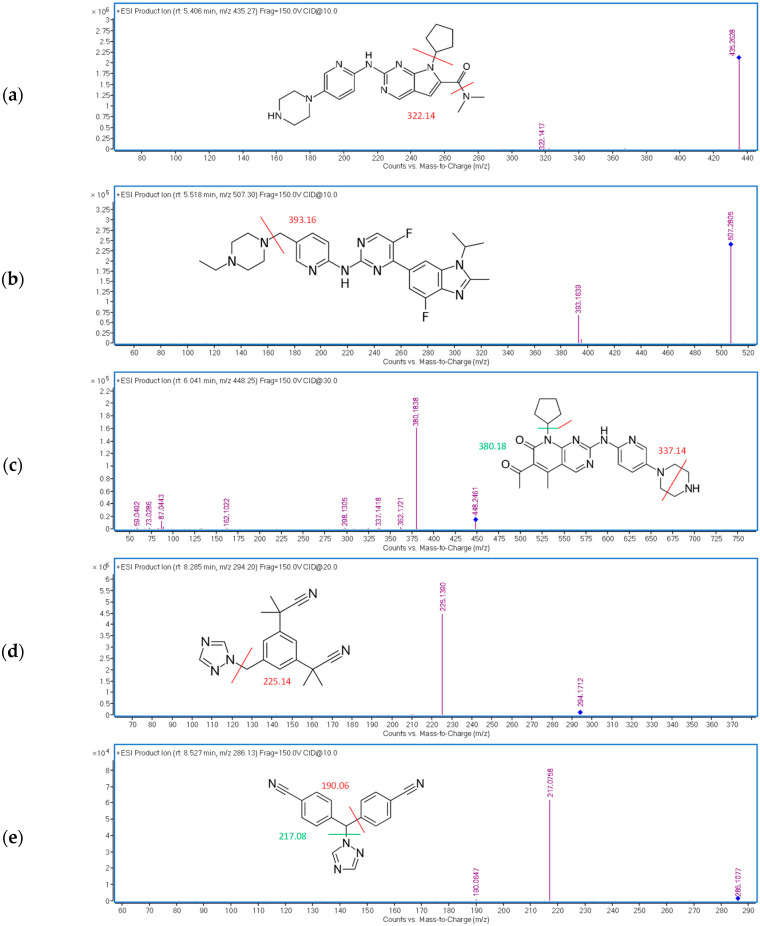

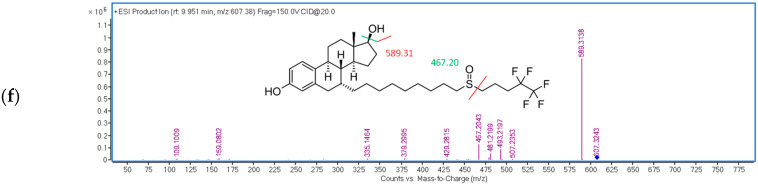
Obtained QTOF-MS/MS mass spectra and proposed fragmentation patterns for all analytes with the optimized collision energies: (**a**) RIB (10 eV), (**b**) ABE (10 eV), (**c**) PAL (30 eV), (**d**) ANA (20 eV), (**e**) LET (10 eV), (**f**) FUL (20 eV).

MS/MS analysis was required to achieve the desired sensitivity and selectivity. Therefore, chromatograms on four collision energies (10–40 eV) were initially recorded and the most abundant fragments of each analyte were detected in the mass spectra. The obtained mass spectra with the proposed fragmentation patterns are shown in Figure 2. Our results are in accordance with the previously described fragmentation patterns [17,19,30]. 

Collision energies which yielded the highest fragment signal intensities were chosen for all analytes, except for RIB. Since RIB is expected in 10–100 times greater concentrations in plasma than the other analytes, a CE of 10 eV was used to achieve minimal fragmentation. Thus, even when a large concentration of the analyte was present in the sample, peak saturation was avoided. 

Mass transition of FUL *m*/*z* 607.32 > 589.31 was associated with an increased background noise, as previously reported [13,31]. Therefore, the transition to *m*/*z* 467.20 was monitored instead. 

A timetable of the collision energies applied according to the analytes’ retention times, along with the corresponding mass transitions, is shown in Table 2. The described conditions were applied over a retention time window of 0.3 min, with an isotopic width of 1.3 *m*/*z*. Data were collected using Agilent MassHunter Workstation software. 

### 2.2. Method Validation

The method was validated according to the EMA and ICH guidelines for bioanalytical method validation [32,33], over a calibration range of 25–5000 ng/mL for RIB, 15–3000 ng/mL for ABE, 3.1–500 ng/mL for PAL, 1–200 ng/mL for ANA, 2.5–500 ng/mL for LET and 5–1000 ng/mL for FUL. Quality control (QC) samples for the precision and accuracy measurements were prepared at four concentration levels: lower limit of quantitation (LLOQ), low, medium, and high QC. Matrix effects and stability were assessed at one lower and one higher concentration level. The following validation parameters were tested: selectivity, carry-over, matrix effect, stability, calibration range, precision and accuracy. Microsoft Office Excel 365 and GraphPad Prism 8 were used for mathematical data analysis.

#### 2.2.1. Selectivity

**Figure 3:**
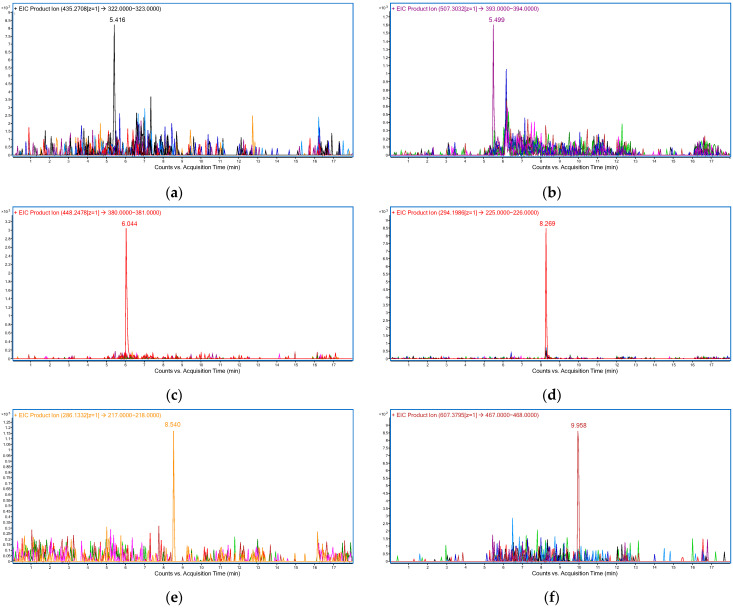
Overlayed EIC of each analyte in plasma at the LLOQ concentration level and EIC of six blank plasmas at the corresponding *m*/*z* transitions: (**a**) RIB (black), (**b**) ABE (purple), (**c**) PAL (red), (**d**) ANA (red), (**e**) LET (orange), (**f**) FUL (brown). In all blank plasma samples, no interferences at the retention times of the analytes were observed above the specified limit.

Selectivity of the method was assessed using plasma from six different sources, including hemolyzed and lipemic plasma. The responses of any interfering components at the retention times of the analytes were not greater than 20% of the analyte response at the LLOQ. The corresponding extracted ion chromatograms (EIC) of six blank plasma samples and plasma samples spiked at the LLOQ concentration levels are shown in Figure 3. 

#### 2.2.2. Carry-Over

Carry-over was assessed during method development by analyzing blank solvent samples after high-concentration plasma samples. In previous studies, the appearance of carry-over has been observed mostly for ABE, PAL and RIB [17,19,22]. Multiple strategies have been proposed for its elimination, from stationary phase exchange [22], different needle wash solvents [17,18,22,34,35], linear range reduction [19] and additional gradient washing steps [17,28,34,35].

In this study, extensive carry-over was observed for ABE, ANA and FUL in up to three subsequent runs following the injections of the highest concentrations of samples. It was successfully eliminated by adding a high flow-rate saw-tooth elution step following each sample run, and a needle wash with 50% MeOH after each injection. Formic acid in water (0.1% *v*/*v*) and 50% MeOH were tested as needle wash solvents. The carry-over observed while using 0.1% formic acid was significantly reduced with 50% MeOH as wash solvent. The responses of any interferences stemming from leftover analytes were less than 20% of the analyte response at the LLOQ. 

#### 2.2.3. Matrix Effect

The matrix effect was tested using blank plasma extracts from six different sources including hemolyzed and lipemic plasma, prepared in triplicate and spiked with all analytes at a lower and higher concentration level after the extraction procedure. It is expressed as mean accuracy (ratio of the peak areas in the presence of matrix and in the absence of matrix) and coefficient of variation (CV) of peak areas between different matrices, as presented in Table 3. The strongest ion suppression was observed for ABE, but it was reproducible between different matrices and concentration levels and was therefore deemed acceptable. Contrary to previous findings [36], the other analytes’ matrix effects were all negligible. The overall low CVs indicate that the matrix effects are concentration- and matrix-independent, and do not significantly affect the analytical accuracy and precision. This may be due to a successful chromatographic separation of the analytes and the interfering matrix components.

#### 2.2.4. Stability

Stability of the stock and working solutions was determined in the preliminary studies. They proved stable for at least eight weeks at 4 °C. Stability of the analytes in plasma was evaluated in terms of processed sample stability on the autosampler for the duration of a typical run (10 °C, 10 h), unprocessed sample stability (room temperature, 2 h), three cycles of freeze–thaw stability (FT, −18 °C to room temperature, thaw duration 30 min) and long term stability at −18 °C for 14 days. Patient plasma samples were stored long-term at −80 °C. Since adequate stability of plasma samples was proven at −18 °C, it was deemed acceptable at lower temperatures as well. Lower- and higher-concentration QC samples were prepared in triplicate for each condition and time point and were analyzed against QC samples freshly prepared in triplicate. The mean bias of percentage of deviation between nominal and measured concentrations and CV should not exceed 15% at any concentration level. The results are shown in Table 4. All analytes proved stable under the tested conditions. 

#### 2.2.5. Linearity and Calibration Range

The linearity of the analyte responses within the set calibration range was confirmed using at least six and up to nine calibration samples. The precision and accuracy of the back-calculated concentrations were within 15% (20% for the LLOQ) for at least 75% and a minimum of six calibration samples. Calibration curves were constructed using linear regression with 1/*x*^2^ weighting. Results of the linearity tests are presented in Table 5. Large between-day CV of the slopes of the calibration curves is a result of the intrinsic systemic variability of the QTOF detector. Therefore, fresh calibration curves had to be prepared daily.

#### 2.2.6. Precision and Accuracy

Precision and accuracy were evaluated using QC samples at four concentration levels prepared in quintuplicate, within one day and between days, against freshly prepared calibration curves. The mean precision (CV between the measurements) and analytical bias (percentage of the difference between the mean measured concentration and the nominal concentration) should not exceed 15% at all calibration levels, and 20% at the LLOQ. All QC samples complied, and the results are reported in Table 6. 

Internal standard (IS) correction is recommended by all guidelines for bioanalytical method validation. The absence of an IS must be technically justified [33].

Due to a large number of analytes and costly reference standards, especially the isotopically labeled counterparts, using the most similar available compounds (excluding drugs that are likely to be used by the patients themselves) was considered. Providing an economically acceptable and still reliable bioanalytical method that could be implemented by financially burdened clinical facilities was one of the priorities of this study. A similar approach was adopted by Al-Shehri et al., who used paracetamol as IS in the LC-MS analysis of RIB, PAL and LET, or Shao et al., whose IS for LET was ANA [37,38]. 

Since none of the drugs from the same therapeutic groups are taken simultaneously by the same patient, and since their structures are inherently analogous, using them as each other’s internal standards was tested. 

For this, separate calibration and QC plasma samples spiked with either RIB, PAL and LET or ABE, ANA and FUL were prepared. Mixtures of ABE and ANA on the one hand, and RIB and LET on the other, were diluted in ACN to appropriate concentrations (around the middle of the calibration range). Protein precipitation was performed using 200 µL of these solutions. Thus, ABE served as IS for RIB and PAL, ANA for LET, RIB for ABE, and LET for ANA and FUL. During the preliminary studies using DAD and FLD, these combinations were promising, but the accuracy and precision on the MS detector did not meet the acceptance criteria set by the validation guidelines. Precision and accuracy were determined using five QC samples per concentration level, on four concentration levels, while matrix effects were evaluated with plasma from two sources, one of which was hemolyzed, on two concentration levels. 

Mean intra-day precision with IS correction (CV) was ≤21.9%, and accuracy (%bias) was ≤28.4% on non-LLOQ concentration levels. Without IS correction, precision and accuracy were ≤15%. Mean CV of matrix effects was 11.4% with IS correction and 7.5% without. The cumulative intra-day CV of IS response was 4.9% for ANA, 6.9% for ABE, 9.9% for LET and 11.2% for RIB (*N* = 20 samples). 

It is evident from these results that the addition of an IS only reduced the method performance. It showed similar behavior to its corresponding analyte to some extent, but could not entirely account for all differences occurring due to matrix effects. However, ion enhancement/suppression effects were reproducible between matrices, even with the simple sample preparation method used. Since adequate precision and accuracy were obtained without the IS and more similar compounds than the ones tested could hardly be found, the method was validated without them. 

### 2.3. Method Application 

This is the first method which provides full chromatographic separation of all six analytes of interest; demonstrates the applicability of three detectors, including a diode array detector, a fluorescence detector and a QTOF MS detector; and is simple, selective and affordable. As opposed to previously published bioanalytical methods, that either did not include all analytes [15,16,17,18,19,20,21,22,37] or did not analyze all six drugs in real patient samples [13], we applied our method to analyze all the drugs of interest in real patients, in a clinical TDM context and for clinical pharmacokinetic evaluation. 

Compared to other previously published methods [15,16,17,18,19,20,22], we developed a simple, fast and reliable LC-MS method for the simultaneous analysis of six drugs used in different therapeutic combinations for breast cancer chemotherapy. A simple sample preparation procedure is applied, and a small amount of plasma is required. Mass detection enabled low quantitation limits and adequate linear ranges for TDM compared to UV detectors [15,16,20]. 

Posocco et al. [17] published a fast, sensitive LC-MS/MS method but limited to only three analytes of interest, PAL, RIB and LET. 

Sato et al. [13] published a fast, accurate and sensitive method with adequate linear ranges for bioanalytical application. However, this method does not provide full chromatographic separation, since ANA, LET and PAL coelute. This can be especially problematic since PAL is used in therapeutic combinations with ANA or LET, so these drugs will inevitably coelute and interfere in real patient samples. Therefore, their method can only be applied if the clinical laboratory possesses a selective detector such as MS. On the other hand, our method provides full chromatographic separation, and can be used with other conventional detectors, such as DAD or FLD, as well as for other analytical purposes. This will enable easier method transfer to all clinical laboratory settings. In this case, a preanalytical concentration step would be advisable.

The carry-over effect, common for the CDK4/6 inhibitors [17,19,22], was apparent on the disclosed blank plasma chromatograms of Sato et al. [13]; however, it was neither reported nor resolved. We also observed this effect and proposed an efficient set of measures for its reduction, including an additional washing step in the mobile phase gradient and a suitable needle-wash solvent. 

Finally, we used a completely different MS detector compared to all other published methods, except for Paul et al. [21]. However, Paul et al. used the QTOF detector without MS/MS fragmentation and the method was applied only to PAL in rat plasma samples. 

#### 2.3.1. Clinical Application to Therapeutic Drug Monitoring

Samples from ten women (ages 35 to 78, median 50.5 years) receiving regular treatment with a combination of a CDK4/6 inhibitor (PAL, RIB or ABE) and endocrine therapy (ANA, LET, FUL or exemestane, EXE) were obtained and analyzed using this LC-MS method. The samples were mostly taken in the elimination phase, during the patients’ regular visit to the hospital before the next dose administration, except in the case of patient 4 (sampling 1 h after dose administration). The aromatase inhibitors and ABE are continuously administered as single daily doses, FUL is a once-monthly intramuscular injection, and the dosing regimen of PAL and RIB involves a week off treatment every 21 days to minimize treatment-induced neutropenia [8]. 

The samples were analyzed against freshly prepared calibration curves. The results are listed in Table 7, while the accompanying chromatograms and mass spectra are included in the Supplementary Materials, Section 1, Figures S1–S29.

Patients receiving LET (*N* = 4) and FUL (*N* = 4) showed minimal inter-individual variabilities (CV 12.2% for LET, 7.1% for FUL) and their concentrations were close to those reported in the clinical studies [26,29]. The patient receiving ANA also had a concentration within the expected range [27]. 

Due to a hypersensitivity reaction to LET, after consulting a clinical pharmacologist, one patient was switched to another aromatase inhibitor, EXE. Therapeutic drug monitoring of EXE has already been assessed in previous clinical studies and it is currently labeled as “not recommended” [14]; therefore, this analyte was not included in this method.

The reported ranges (Cmin to Cmax) of ABE and RIB concentrations in plasma are exceptionally wide [24,25]. A minimal threshold concentration for efficacy of ABE proposed in reported PK/PD studies was 200 ng/mL [39]. The patient receiving ABE in our study had a plasma concentration above this value. 

The patients receiving RIB (*N* = 6) can be divided into several subcategories according to the sampling time: 1 h after dose administration (*N* = 1), trough concentration at steady state (*N* = 2) and in the elimination phase during the week off (*N* = 3). Concentrations of RIB shortly after administration have been reported [40], and the levels measured in this work are comparable, as well as those determined in the steady state.

The low RIB and PAL levels detected in patient samples 2, 6–10 are due to sampling taking place during the off-treatment week. The majority of regular patient visits to the hospital are scheduled during this phase. It is important to note that additional sampling outside of the regular visiting schedule may prove inconvenient for patients. Certain inter-individual variability can be observed among these patients, although some of it may stem from the concentrations being near the LOD. Generally, for therapeutic drug monitoring, samples representing the trough concentration (plasma concentration in the elimination phase during the steady state) are considered the most valuable. Concentrations measured after a week off treatment can be informative as to how successfully a drug is eliminated by each patient and thus how efficiently toxicity is managed. 

Patient 10, sampled only two days after the end of the therapy cycle, showed no trace of PAL in plasma. This patient was previously switched from RIB to PAL due to severe side effects; therefore, it is very likely that the result observed could be due to reduced adherence to the treatment regimen. Patients 3 and 5 received the same CDK4/6 inhibitors and the sampling was carried out for both patients in the steady state. However, their concentrations significantly differ, which could potentially be a consequence of differences in patients’ characteristics or nonadherence. Our clinical experience suggests that women with lower body mass index exhibit stronger dose-dependent toxicity in the third week of the treatment cycle. Some patients, without seeking medical approval, intentionally reduce or skip the recommended dose to minimize these adverse effects. All of this supports the notion that the one-size-fits-all concept of treatment is not applicable in all cases, and there is a potential benefit from tailoring treatment to an individual patient’s needs. This may be achieved by appropriately applying TDM to guide the dose adjustment for patients taking these medications.

#### 2.3.2. Application to Clinical Pharmacokinetic Evaluation

**Figure 4:**
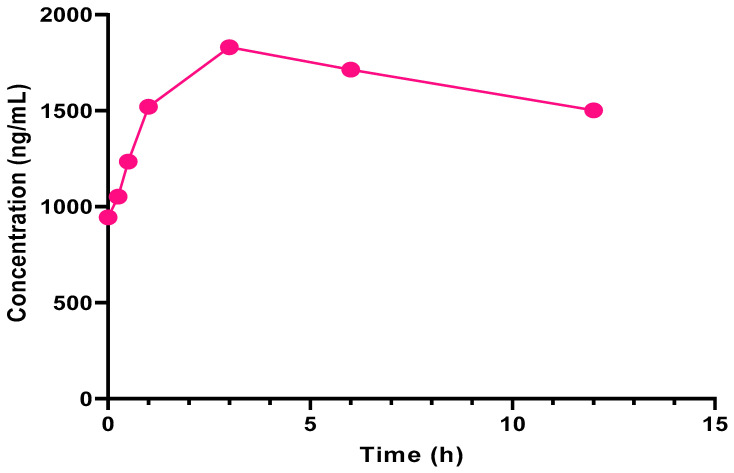
Plasma concentration vs. time curve for RIB following the administration of a 600 mg oral dose.

Finally, we applied our fully validated method to evaluate the pharmacokinetic disposition of RIB in a patient during the first day of a treatment cycle. The plasma samples were prepared in the same manner as the single-point study samples and quantitated using a freshly prepared calibration curve. No additional dilution or preconcentration was necessary, as all the determined concentrations fell within the validated range. The obtained plasma concentration vs. time curve for RIB is shown in Figure 4. The corresponding data are available in Table S1 of the Supplementary Materials, Section 2 (Results of the pharmacokinetic study).

Pharmacokinetic data were analyzed with the PK solver Excel extension, using the linear trapezoidal model for non-compartmental analysis of plasma data after extravascular input. The maximum concentration (Cmax, 1830 ng/mL) was achieved three hours after dose administration. The beginning of the elimination phase was estimated around three hours after dosing. Ribociclib is administered as an instant release tablet dosage form, so no absorption lag was neither expected nor observed. The calculated elimination constant was 0.022 h^−1^, while the elimination half-life was 31.55 h. The obtained results are in accordance with the data previously reported in the clinical trials (a Cmax of 1510 to 2790 ng/mL was mostly achieved between 1 and 4 h, with the mean terminal half-life between 29.7 and 54.7 h) [39].

The successful application of this method for the quantitation of both single-point samples and pharmacokinetic study samples demonstrates its suitability for use in clinical studies involving a larger number of patients. This is particularly important in personalized medicine. Measuring the drug levels in the clinic will allow the clinician to tailor the dose to an individual patient’s drug exposure and therapeutic response, thereby minimizing the risk of adverse effects and drug–drug interactions.

## 3. Materials and Methods

### 3.1. Chemicals and Reagents

ACN (HPLC and LC-MS grade) and MeOH (HPLC grade) were purchased from J. T. Baker (Phillipsburg, NJ, USA). Water was purified using a Merck Mili-Q IQ 7000 water purification system (Darmstadt, Germany). Formic acid (for LC-MS) was obtained from Supelco (Bellefonte, PA, USA), glacial acetic acid (purity > 99.7%) was from Panreac (Barcelona, Spain), ammonium acetate (purity > 98%) was from Lach:Ner (Neratovice, Czech Republic) and ammonium bicarbonate (purity > 99%) was from Sigma-Aldrich (St. Gallen, Switzerland). Standards of ANA and LET (purity > 98%) were purchased from Tokyo Chemical Industry (Tokyo, Japan), FUL (purity > 97%) from Sigma-Aldrich (St. Gallen, Switzerland), PAL and ABE (purity > 98%) from Toronto Research Chemicals (Toronto, Canada), and RIB (purity > 98%) from BioVision (San Francisco, CA, USA).

### 3.2. Stock and Working Solutions

Calibration and quality control standards were prepared from separate stock solutions. ABE, RIB, ANA, LET and FUL stock solutions were prepared at 1 mg/mL in MeOH. Stock solution of PAL was prepared at 0.25 mg/mL in 50% ACN. Working solutions were prepared by serial dilution of the stock solutions to nine concentration levels for calibration standards (Table 8), four QC concentration levels for precision and accuracy tests and two QC concentration levels for matrix effect and stability assessments. The stock and working solutions were stored at 4 °C. Each working solution was diluted 10-fold with blank plasma prior to analysis to obtain the calibration and QC plasma samples.

### 3.3. Blank and Patient Plasma

The developed analytical method used for the analysis of human plasma samples was approved by the Ethics Committee of University of Zagreb Faculty of Pharmacy and Biochemistry (approval number 251-62-03-19-30) and by the Ethics Committee of University Hospital Centre Zagreb (approval number 02/21-JG).

Healthy and patient blood samples were collected and pre-treated at the University Hospital Centre Zagreb, with signed informed consent. Patients receiving a combination of a CDK4/6 inhibitor and endocrine therapy as regular treatment for at least 4 months were enrolled.

Whole blood was collected in containers with K_2_-EDTA anticoagulant and centrifuged for 10 min at 1500 g; the supernatant was then withdrawn and stored at −18 °C (blank plasma) or −80 °C (patients’ plasma). Hemolyzed plasma was obtained by briefly freezing a whole blood sample at −18 °C before continuing with the procedure.

### 3.4. Sample Preparation Procedure

Calibration and QC samples were prepared by diluting 5 µL of the appropriate working solutions of the analytes with 45 µL of blank plasma in 1.5 mL polypropylene centrifuge tubes. In case of patient sample analysis, an aliquot of 50 µL of patient plasma was used. Proteins were precipitated by adding 200 µL of ACN, after which the samples were vortex-mixed for 10 s and centrifuged at 2000 g for 5 min. The resulting supernatant was collected and 200 µL was evaporated to dryness in a vacuum evaporator at room temperature. The dry residue was dissolved in 40 µL of 65% methanol, while 5 µL was injected into the chromatographic system.

### 3.5. Instruments and Software

An Agilent 1100 HPLC equipped with DAD and FLD was employed in the preliminary studies; the LC-MS analyses were performed on a 1290 UHPLC coupled to a 6550 iFunnel QTOF MS with a Jet Stream ESI source (Agilent, Santa Clara, CA, USA). The mobile phase gradient and flow rates are depicted in Table 9.

An Agilent Dual Jet Stream electrospray (ESI) source was used in positive ionization mode with nitrogen as drying, nebulizer and sheath gas. Parameters such as the sheath gas temperature, ESI source and nozzle voltages, TOF fragmentor voltage, collision energies and mass acquisition rates were optimized to achieve the best analyte responses in the desired concentration ranges. The following final conditions were applied: drying gas temperature 200 °C; drying gas flow 14 L/min; nebulizer pressure 35 psi; sheath gas flow 11 L/min. TOF mass calibration was performed daily in the extended dynamic range (2 GHz) and low mass range (1700 *m*/*z*). The reference masses used for the within-run mass correction were *m*/*z* 121.0509 and 922.0098.

Data were collected using an Agilent MassHunter Workstation software Qualitative analysis 10.0 and Data Acquisition for 6200 series TOF/6500 series QTOF 10.1 (Santa Clara, CA, USA). Microsoft Office 365 Excel (Redmond, WA, USA) with the PK solver extension and GraphPad Prism 8 (San Diego, CA, USA) were used for data analysis.

## 4. Conclusions

A novel sensitive and selective LC-MS method for the simultaneous analysis of PAL, RIB, ABE, ANA, LET and FUL was developed, validated and applied to patient plasma samples. The MS detection conditions were optimized to achieve linear ranges adjusted to each analytes’ expected plasma levels, as previously reported in clinical trials.

A simple sample preparation method was employed, using only 50 µL of plasma, comprising protein precipitation and solvent evaporation. Matrix effects were consistent between matrices and different analyte concentrations, with most ion suppression observed for ABE.

All analytes eluted within 10 min. The intra-day method precision (CV) ranged from 3.1% to 15%, while intra-day accuracy (%bias) was between −1.5% and 15.0%. The inter-day precision ranged from 1.6% to 14.9%, with accuracy between −14.3% and 14.6%, which is in accordance with the EMA and ICH guidelines on bioanalytical method validation.

Although carry-over was observed, it was resolved by using an additional high flow-rate saw-tooth step in the gradient and applying needle wash with 50% MeOH after each injection.

This is the first method that provides full chromatographic separation of all six analytes of interest and demonstrates the applicability of three detectors, including a diode array detector, a fluorescence detector and a QTOF mass spectrometer. Furthermore, compared to previously published bioanalytical methods, this method was applied for the quantitation of all drugs of interest in real patients in a clinical TDM context, as well as for clinical pharmacokinetic evaluation. This demonstrates the suitability of the method for use in the clinical environment as a prerequisite for personalized breast cancer treatment. Therapeutic drug monitoring of these drugs will allow clinicians to tailor the dose to an individual patient’s drug exposure and therapeutic response, hence minimizing the risk of toxicity and of drug–drug interactions.

## Figures and Tables

**Table 1:** Limits of detection at the optimized conditions and expected plasma concentrations obtained from literature (ng/mL).

Analyte	RIB	ABE	PAL	ANA	LET	FUL
LOD ^1^ DAD	30	40	120	1600	140	6190
LOD FLD	ND	ND	ND	900	220	600
LOD MS	20	8	1	<0.5 ^2^	1	1.5
Expected literature range C_min_–C_max_	711–3500 [24]	102.65–1381 [25]	72.8–185.5 [26]	22–52.6 [27]	28.4–349.2 [28]	16–25 [29]

^1^ LODs were estimated from signal/noise ratios of spiked plasma samples serially diluted with blank plasma as the concentration at which the signal is three times greater than the surrounding noise. ^2^ The lowest analyzed concentrations of ANA using MS detector (0.5 ng/mL) yielded signal/noise values of ≈50. ND—not determined.

**Table 2:** Analytes’ chromatographic retention times, optimized collision energies (CE) and mass transitions used for quantitation.

Analyte	t_R_ (min)	CE (eV)	*m*/*z* Transition
RIB	5.4	10	435.27 → 322.14
ABE	5.5	10	507.30 → 393.16
PAL	6.2	30	448.25 → 380.18
ANA	8.2	20	294.20 → 225.14
LET	8.5	10	286.13 → 217.08
FUL	9.9	20	607.38 → 467.20

**Table 3:** Mean matrix effect and CV of analyte responses across different plasma sources (*N* = 3 samples per plasma source and concentration level).

Analyte	Concentration (ng/mL)	Matrix Effect (%)	CV (%)	Analyte	Concentration (ng/mL)	Matrix Effect (%)	CV (%)
RIB	62.5	108.34	3.15	ANA	2.5	90.36	6.85
	2500	102.26	2.14		100	106.78	3.69
ABE	37.5	77.80	6.69	LET	6.25	83.81	9.94
	1500	73.10	2.47		250	101.67	6.97
PAL	6.25	115.74	5.70	FUL	12.5	92.78	8.92
	250	106.74	4.08		500	101.08	6.90

**Table 4:** Stability results at the tested conditions (*N* = 3 samples per concentration level and time point).

Analyte	Concentration (ng/mL)		Bench Top (2 h, 25 °C)	Auto- Sampler (10 h, 10 °C)	1 FT Cycle (30 min, −18–25 °C)	2 FT Cycles (30 min, −18–25 °C)	3 FT Cycles (30 min, −18–25 °C)	Long-Term (2 Weeks, −18 °C)
RIB	62.5	Bias (%)	6.01	8.95	7.88	1.69	6.00	13.89
		CV (%)	5.07	9.50	12.89	11.16	5.02	14.77
	2500	Bias (%)	4.96	−6.47	11.10	−1.66	−4.69	−10.69
		CV (%)	1.69	5.18	7.05	7.05	1.61	4.45
ABE	37.5	Bias (%)	9.09	11.51	13.23	−7.43	14.07	7.60
		CV (%)	8.04	7.41	3.47	6.89	2.55	7.84
	1500	Bias (%)	−0.10	14.88	9.37	5.68	1.13	3.09
		CV (%)	3.82	4.25	1.56	1.56	6.93	5.21
PAL	6.2	Bias (%)	3.08	13.69	14.97	1.86	2.81	12.29
		CV (%)	10.27	1.35	4.99	6.62	4.84	9.95
	250	Bias (%)	−4.37	14.95	6.35	−1.65	−7.90	−3.08
		CV (%)	3.35	5.32	5.95	5.95	3.84	10.44
ANA	2.5	Bias (%)	−10.33	2.27	−7.88	−2.80	−3.98	3.09
		CV (%)	5.01	11.15	1.93	4.74	5.32	3.87
	100	Bias (%)	−0.62	4.89	6.65	8.39	−0.69	−0.40
		CV (%)	2.13	2.60	2.78	2.78	6.48	8.54
LET	6.25	Bias (%)	−6.81	6.11	−0.06	−6.52	−3.50	−10.44
		CV (%)	8.64	7.01	14.71	5.53	6.79	2.90
	250	Bias (%)	6.16	−0.29	2.07	−0.26	−7.61	−7.75
		CV (%)	1.66	2.95	2.80	2.80	9.51	8.45
FUL	12.5	Bias (%)	−9.36	7.38	−14.59	−13.24	−8.90	−5.01
		CV (%)	14.83	−3.68	10.68	14.99	4.62	9.00
	500	Bias (%)	2.32	−8.06	2.23	4.49	−7.51	−1.62
		CV (%)	1.74	1.37	8.71	8.71	7.33	4.46

**Table 5:** Calibration ranges and regression parameters for all analytes (*N* = 6–9 calibration samples per day).

	Analyte	RIB	ABE	PAL	ANA	LET	FUL
	Range (ng/mL)	25–5000	15–3000	3.1–500	1–200	2.5–500	5–1000
Within-day ^1^	Slope	84.48	500.67	6194.67	27,818.67	1235.33	664.97
	CV (%)	3.94	2.00	5.68	4.77	4.93	4.97
	Intercept	875.13	−246.27	−2113.33	1840.33	356.07	452.73
Between-day ^2^	Slope	78.44	459.75	5940.75	25,972.75	1350.25	513.63
	CV (%)	7.52	40.23	10.49	15.52	9.75	27.09
	Intercept	771.03	2342.95	−3629.25	8426.75	576.38	627.63
	R	0.9941–0.9985	0.9955–0.9983	0.9950–0.9991	0.9958–0.9990	0.9931–0.9996	0.9953–0.9984

^1^ Within-day—based on three calibration curves constructed on the same day. ^2^ Between-day—based on four calibration curves constructed in four days.

**Table 6:** Intra- and inter-day precision and accuracy (*N* = 5 samples per concentration level).

		Intra-Day		Inter-Day	
Analyte	Concentration (ng/mL)	Bias (%)	CV (%)	Bias (%)	CV (%)
RIB	25	−1.8	11.5	−14.7	19.9
	50	6.4	8.9	7.0	11.7
	2000	8.5	3.7	−0.1	7.9
	3750	−1.5	3.7	−7.2	8.0
ABE	15	4.2	19.4	−7.8	17.4
	30	2.2	14.8	−6.8	4.1
	1200	12.3	14.6	4.4	3.2
	2250	14.9	3.1	−12.9	5.0
PAL	3.1	15.4	7.1	9.5	7.9
	6.2	10.3	6.4	9.2	8.9
	250	15.0	4.6	3.1	5.1
	468.8	11.1	6.0	−0.3	4.3
ANA	1	8.3	3.7	−0.5	6.1
	2	4.6	9.6	3.3	6.0
	80	0.1	14.3	−5.4	3.9
	150	5.4	6.2	−3.7	1.6
LET	2.5	14.5	3.7	−14.8	16.4
	5	5.8	11.5	14.6	6.9
	200	6.8	7.1	4.0	7.9
	375	4.2	5.1	−4.9	6.0
FUL	5	3.3	16.2	−2.7	11.9
	10	11.6	14.9	−14.3	13.9
	400	4.6	15.0	−6.3	5.4
	750	−1.1	7.2	2.2	14.9

**Table 7:** Drug concentrations in patient plasma samples.

Patient	Sampling Day ^1^	CDK4/6 Inhibitor	Plasma Concentration (ng/mL)	Endocrine Agent	Plasma Concentration (ng/mL)
1	NA	ABE (300 mg daily)	425.79	LET (2.5 mg daily)	109.99
2	day 24	RIB (600 mg daily)	57.43	LET (2.5 mg daily)	141.11
3	day 16	RIB (600 mg daily)	682.16	LET (2.5 mg daily)	146.21
4	day 1	RIB (600 mg daily)	1519.81	LET (2.5 mg daily)	139.46
5	day 12	RIB (600 mg daily)	1192.77	ANA (1 mg daily)	43.96
6	day 28	RIB (600 mg daily)	27.18	EXE (25 mg daily)	ND
7	day 27	RIB (600 mg daily)	40.67	FUL (500 mg monthly)	20.73
8	day 27	PAL (125 mg daily)	7.55	FUL (500 mg monthly)	22.69
9	day 28	PAL (125 mg daily)	<LOQ	FUL (500 mg monthly)	22.13
10	day 23	PAL (125 mg daily)	<LOD	FUL (500 mg monthly)	24.56

^1^ According to the PAL/RIB dosing cycle. NA—not applicable, ABE is administered continuously.

**Table 8:** Final concentration levels of the calibration plasma samples (ng/mL).

	RIB	ABE	PAL	ANA	LET	FUL
1	25	15	2.5	1	2.5	5
2	50	30	5	2	5	10
3	125	75	12.5	5	12.5	25
4	250	150	25	10	25	50
5	750	450	75	30	75	150
6	1500	900	150	60	150	300
7	2500	1500	250	100	250	500
8	3750	2250	375	150	375	750
9	5000	3000	500	200	500	1000

**Table 9:** Mobile phase gradient composition and flow rates.

t (min)	% B	Flow Rate (mL/min)
0	15	0.4
8.5	100	0.4
10	100	0.4
10.5	15	0.4
11.3	100	1
13	100	1
13.2	15	1
13.5	15	0.4
18	15	0.4

## Data Availability

Data is contained within the article and supplementary material.

## Supplementary Materials

The following supporting information can be downloaded at: https://www.mdpi.com/article/10.3390/ph15050614/s1, Section 1: Chromatograms and MS/MS spectra of the analyzed single-point patient samples: Figure S1: EIC of ABE (transition *m*/*z* 507.3→393.2) and LET (transition *m*/*z* 286.1→217.1); Figure S2: Mass spectrum of peak 1 (ABE, tr 5.524 min); Figure S3: Mass spectrum of peak 2 (LET, tr 8.542 min); Figure S4: EIC of RIB (transition *m*/*z* 435.3→322.1) and LET (transition *m/z* 286.1→217.1); Figure S5: Mass spectrum of peak 1 (RIB, tr 5.414 min); Figure S6: Mass spectrum of peak 2 (LET, tr 8.545 min); Figure S7: EIC of RIB (transition *m/z* 435.3→322.1) and LET (transition *m/z* 286.1→217.1); Figure S8: Mass spectrum of peak 1 (RIB, tr 5.411 min); Figure S9: Mass spectrum of peak 2 (LET, tr 8.544 min); Figure S10: EIC of RIB (transition *m/z* 435.3→322.1) and LET (transition *m/z* 286.1→217.1); Figure S11: Mass spectrum of peak 1 (RIB, tr 5.412 min); Figure S12: Mass spectrum of peak 2 (LET, tr 8.546 min); Figure S13: EIC of RIB (transition *m/z* 435.3→322.1) and ANA (transition *m/z* 294.2→225.1); Figure S14: Mass spectrum of peak 1 (RIB, tr 5.415 min); Figure S15: Mass spectrum of peak 2 (ANA, tr 8.273 min); Figure S16: EIC of RIB (transition *m/z* 435.3→322.1); Figure S17: Mass spectrum of the peak (RIB, tr 5.416 min); Figure S18: EIC of RIB (transition *m/z* 435.3→322.1) and FUL (transition *m/z* 607.4→467.2); Figure S19: Mass spectrum of peak 1 (RIB, tr 5.420 min); Figure S20: Mass spectrum of peak 2 (FUL, tr 9.977 min); Figure S21: EIC of PAL (transition *m/z* 448.3→380.2) and FUL (transition *m/z* 607.4→467.2); Figure S22: Mass spectrum of peak 1 (PAL, tr 6.065 min); Figure S23: Mass spectrum of peak 2 (FUL, tr 9.960 min); Figure S24: EIC of PAL (transition *m/z* 448.3→380.2) and FUL (transition *m/z* 607.4→467.2); Figure S25: Mass spectrum of peak 1 (PAL, tr 6.052 min); Figure S26: Mass spectrum of peak 2 (FUL, tr 9.976 min); Figure S27: EIC of PAL (transition *m/z* 448.3→380.2) and FUL (transition *m/z* 607.4→467.2); Figure S28: Mass spectra around the retention time of PAL, 6.0 min; Figure S29: Mass spectrum of the peak (FUL, tr 9.977 min); Section 2: Results of the pharmacokinetic study: Table S1: Exact RIB plasma concentrations measured at each timepoint.
