# Supplementary material for: Development and Validation of a Novel LC-MS/MS Method for the Simultaneous Determination of Abemaciclib, Palbociclib, Ribociclib, Anastrozole, Letrozole, and Fulvestrant in Plasma Samples: A Prerequisite for Personalized Breast Cancer Treatment
Source: Pharmaceuticals (Basel). 2022 May 16;15(5):614. doi: 10.3390/ph15050614 (PMC9147789; doi:10.3390/ph15050614)
Supplement: Supplementary file 1 [file pharmaceuticals-15-00614-s001.zip › pharmaceuticals-1691778-supplementary.pdf]

Supplementary materials

Development and Validation of a Novel LC-MS/MS Method for the Simultaneous Determination of Abemaciclib, Palbociclib, Ribociclib, Anastrozole, Letrozole, and Fulvestrant in Plasma Samples: A Prerequisite for Personalized Breast Cancer Treatment

Lu Turković [1], Luka Bočkor [2], Oscar Ekpenyong [3], Tajana Silovski [4], Mila Lovrić [5,6], Slaven Crnković [7], Biljana Nigović [1] and Miranda Sertić [1,*]

1 Department of Pharmaceutical Analysis, Faculty of Pharmacy and Biochemistry, University of Zagreb, Ante Kovacica 1, 10000 Zagreb, Croatia; lu.turkovic@pharma.unizg.hr (L.T.); bnigovic@pharma.hr (B.N.)
2 Centre for Applied Bioanthropology, Institute for Anthropological Research, Ljudevita Gaja 32, 10000 Zagreb, Croatia; lbockor@inantro.hr
3 ADME & Discovery Toxicology, Merck & Co., Inc., South San Francisco, CA 94080, USA; oscar.ekpenyong@merck.com
4 Department of Oncology, University Hospital Centre Zagreb, Kispaticeva 12, 10000 Zagreb, Croatia; tsilovsk@kbc-zagreb.hr
5 Department of Laboratory Diagnostics, University Hospital Centre Zagreb, Kispaticeva 12, 10000 Zagreb, Croatia; mila.lovric@kbc-zagreb.hr
6 Faculty of Pharmacy and Biochemistry, University of Zagreb, Ante Kovacica 1, 10000 Zagreb, Croatia; mila.lovric@kbc-zagreb.hr
7 Division of Physiology, Medical University of Graz, Ludwig Boltzmann Institute for Lung and Vascular Research, Neue Stiftingtalstrasse 6/V, 8010 Graz, Austria; slaven.crnkovic@medunigraz.at
* Correspondence: miranda.sertic@pharma.unizg.hr; Tel.: +385-1-6394-473

Section 1. Chromatograms and MS/MS spectra of the analyzed single-point patient samples:

Patient 1 (ABE + LET)

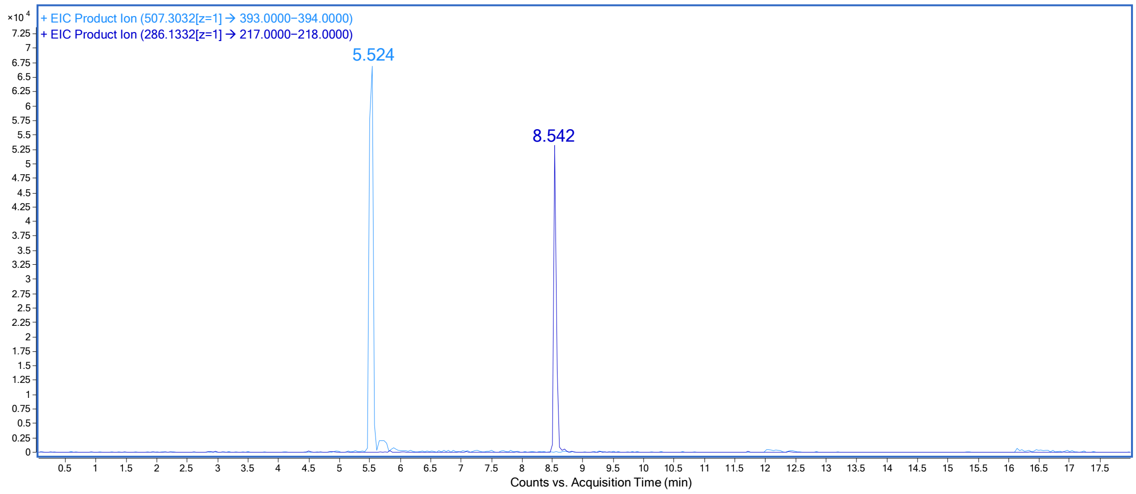

Figure S1. EIC of ABE (transition *m/z* 507.3→393.2) and LET (transition *m/z* 286.1→217.1).

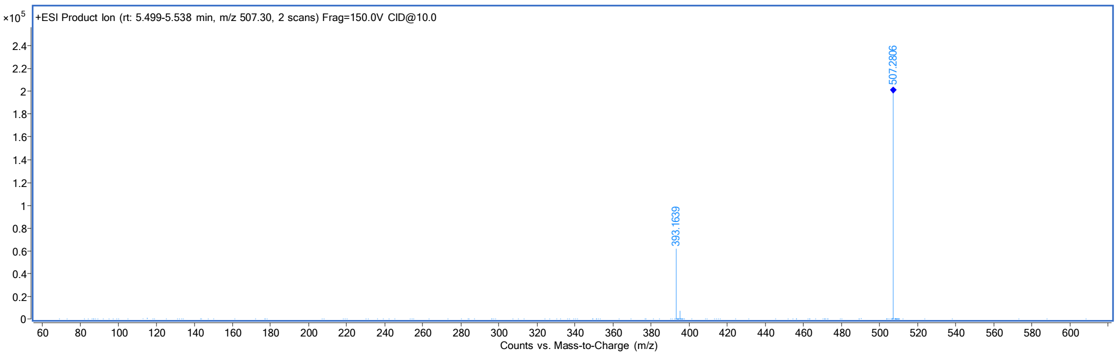

Figure S2. Mass spectrum of peak 1 (ABE, tr 5.524 min).

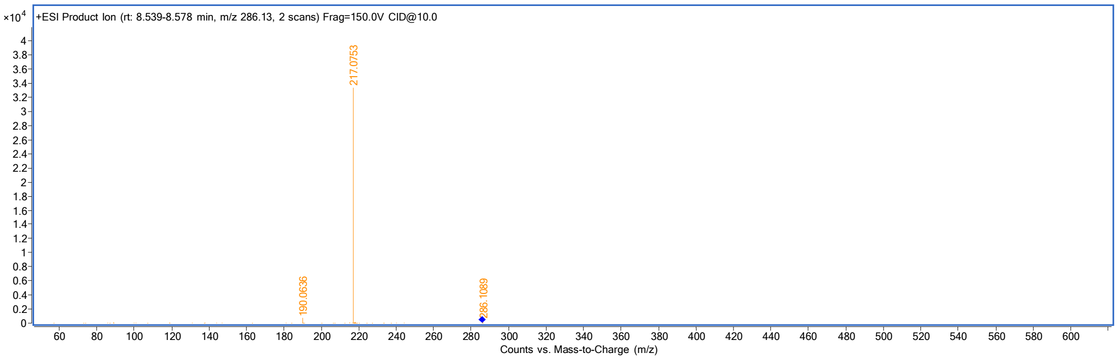

Figure S3. Mass spectrum of peak 2 (LET, tr 8.542 min).

Patient 2 (RIB + LET)

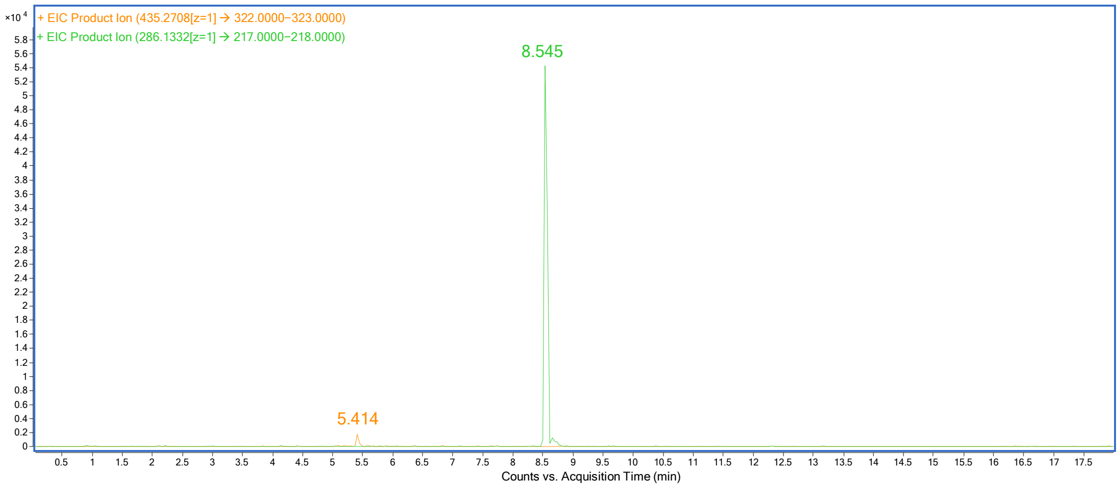

Figure S4. EIC of RIB (transition *m/z* 435.3→322.1) and LET (transition *m/z* 286.1→217.1).

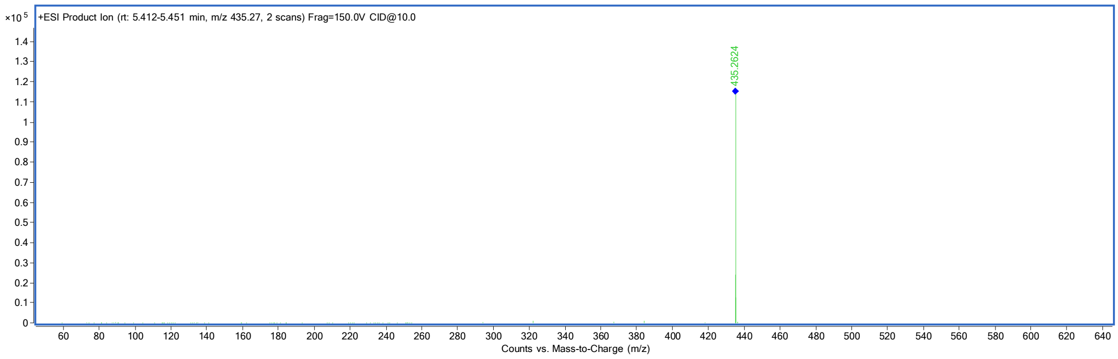

Figure S5. Mass spectrum of peak 1 (RIB, tr 5.414 min).

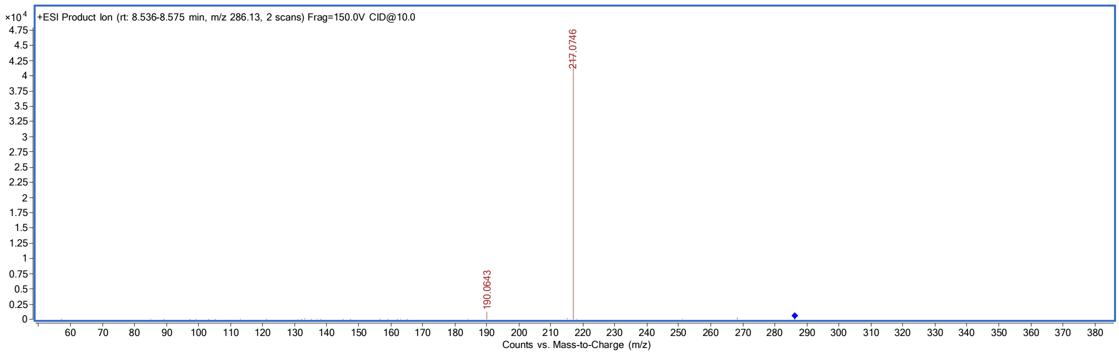

Figure S6. Mass spectrum of peak 2 (LET, tr 8.545 min).

Patient 3 (RIB + LET)

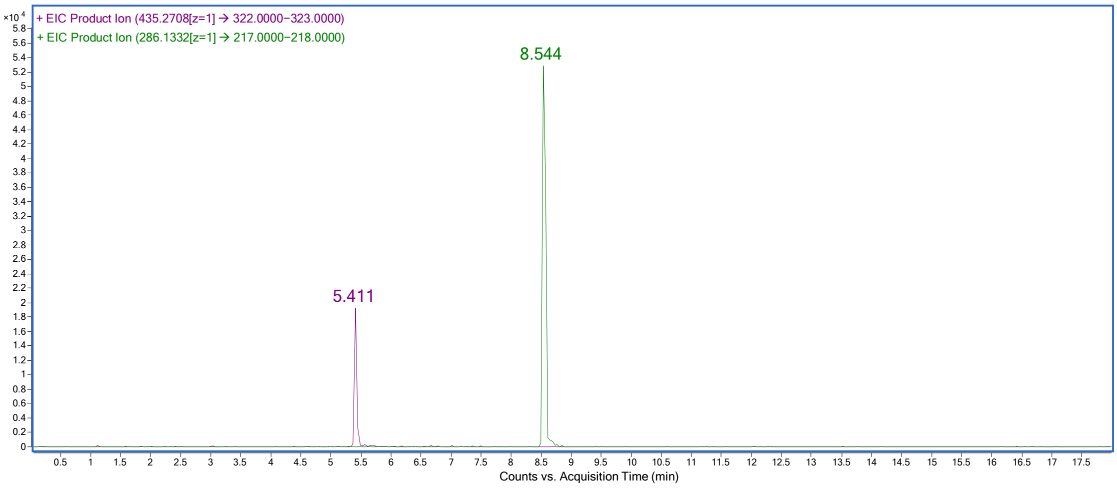

Figure S7. EIC of RIB (transition *m/z* 435.3→322.1) and LET (transition *m/z* 286.1→217.1).

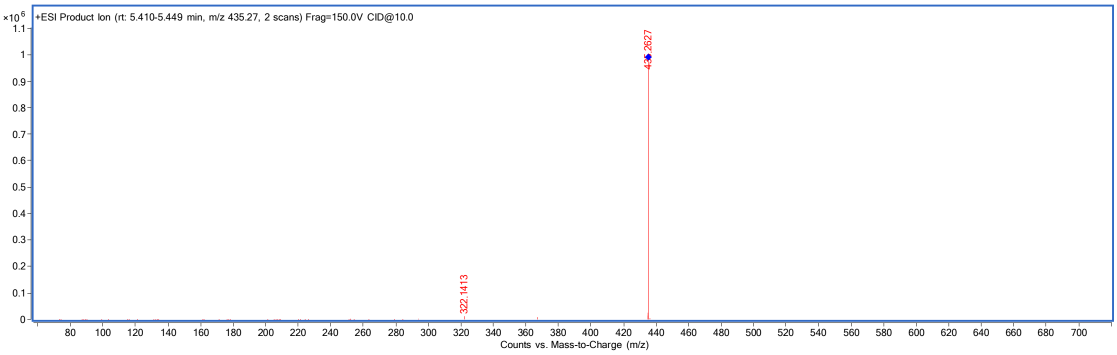

Figure S8. Mass spectrum of peak 1 (RIB, tr 5.411 min).

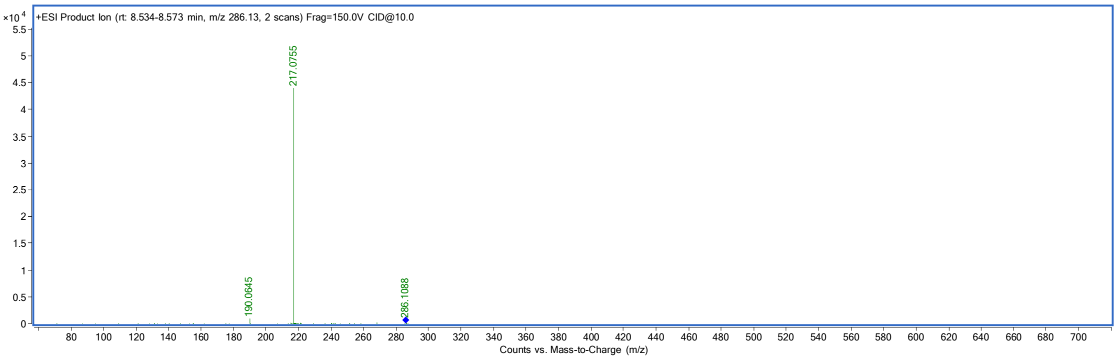

Figure S9. Mass spectrum of peak 2 (LET, tr 8.544 min).

Patient 4 (RIB + LET)

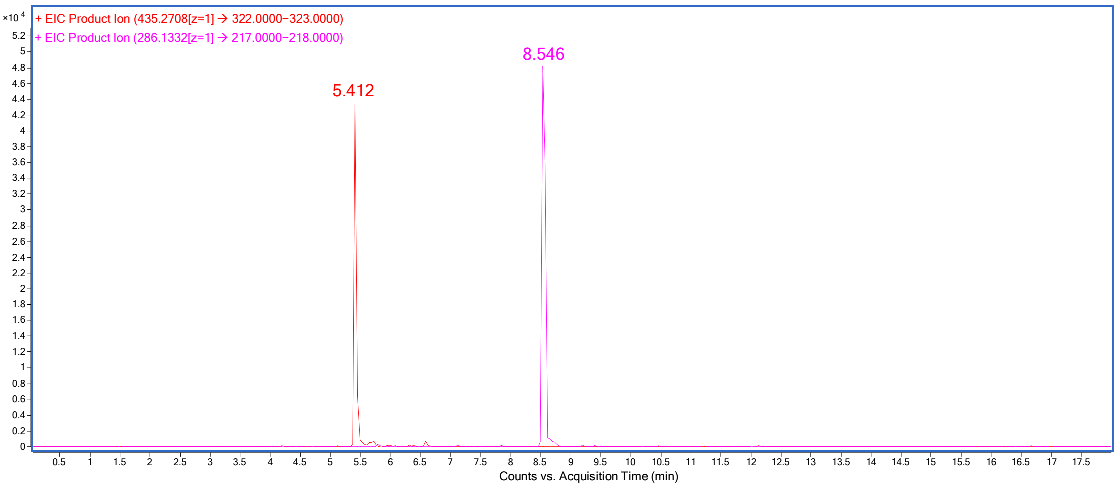

Figure S10. EIC of RIB (transition *m/z* 435.3→322.1) and LET (transition *m/z* 286.1→217.1).

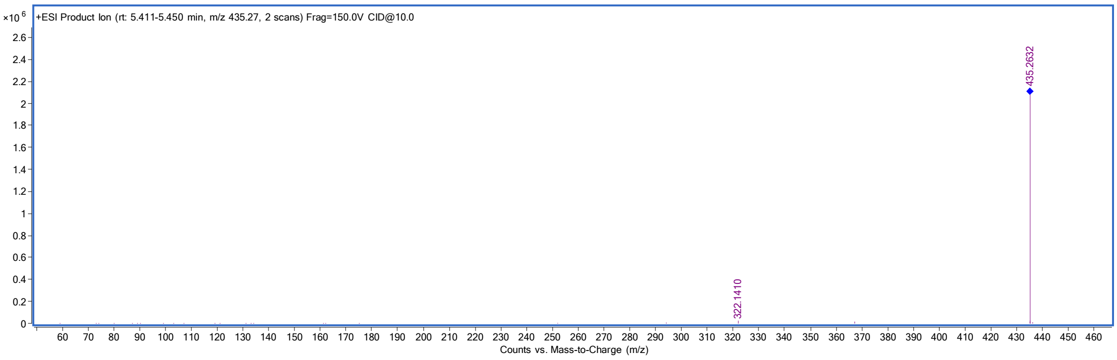

Figure S11. Mass spectrum of peak 1 (RIB, tr 5.412 min).

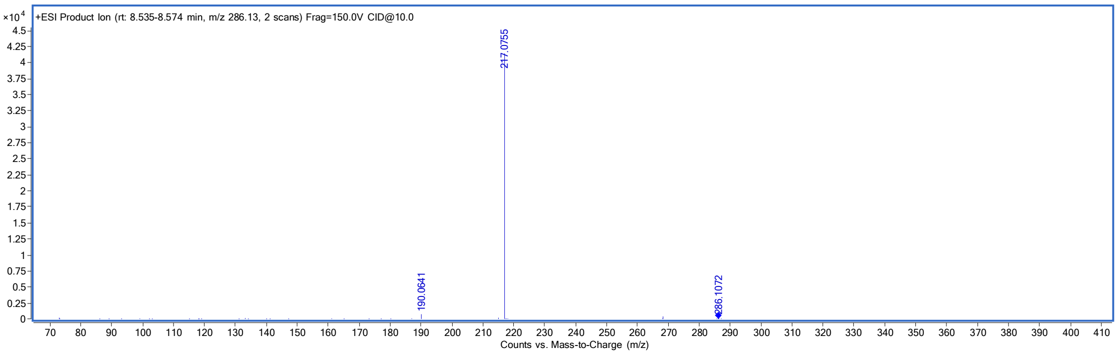

Figure S12. Mass spectrum of peak 2 (LET, tr 8.546 min).

Patient 5 (RIB + ANA)

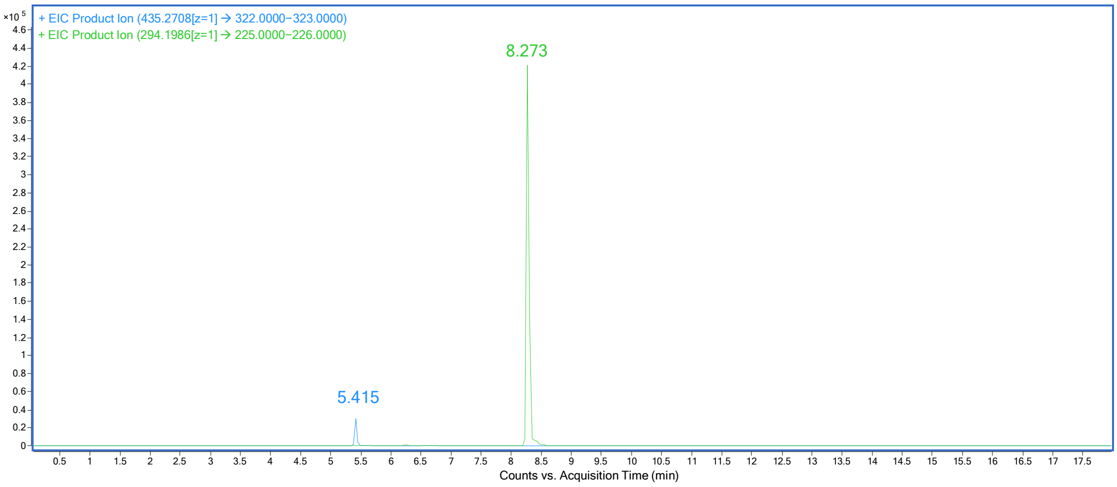

Figure S13. EIC of RIB (transition *m/z* 435.3→322.1) and ANA (transition *m/z* 294.2→225.1).

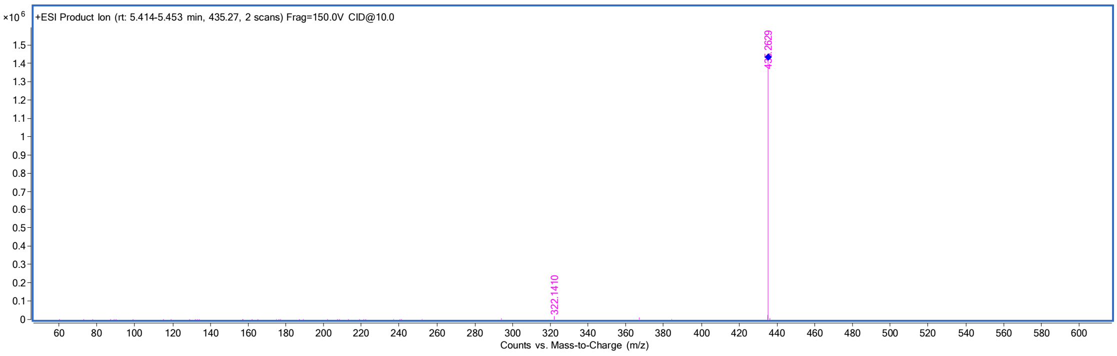

Figure S14. Mass spectrum of peak 1 (RIB, tr 5.415 min).

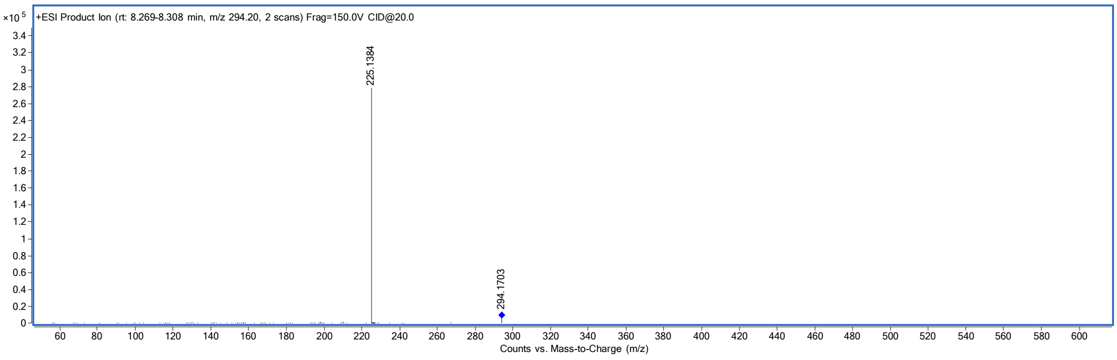

Figure S15. Mass spectrum of peak 2 (ANA, tr 8.273 min).

Patient 6 (RIB + EXE)

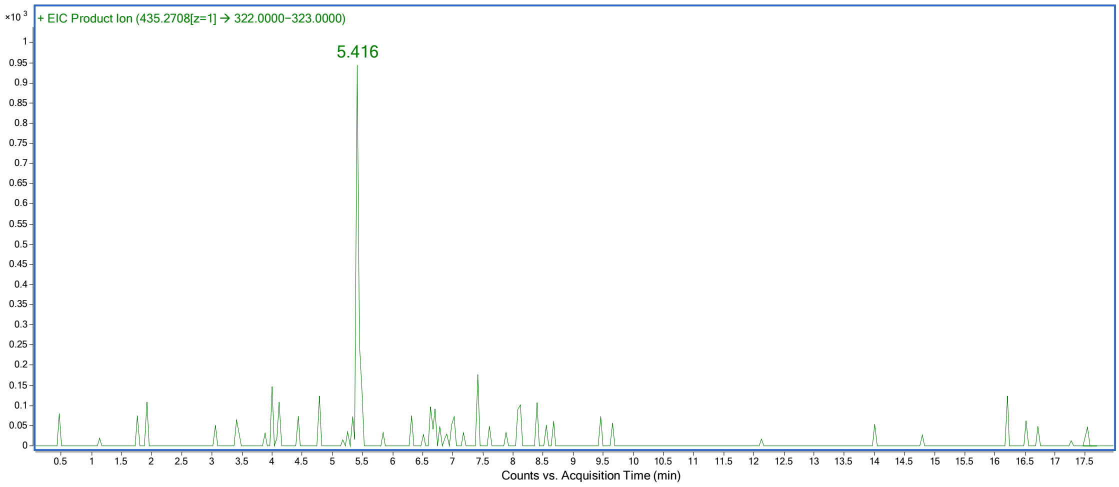

Figure S16. EIC of RIB (transition *m*/*z* 435.3→322.1).

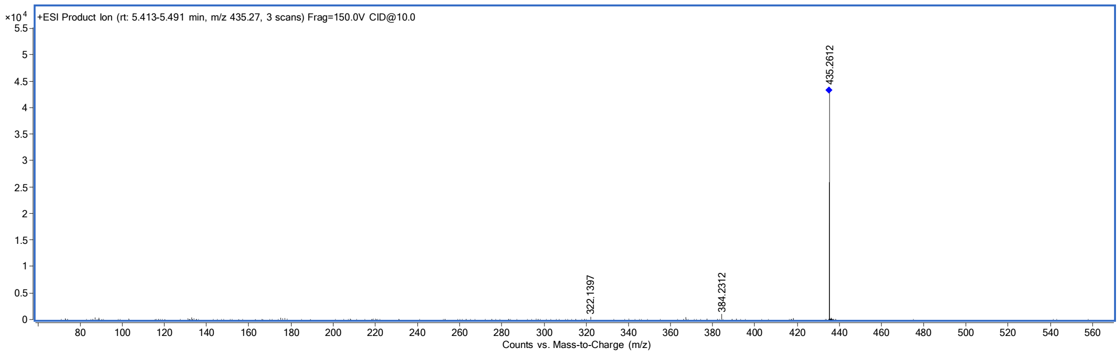

Figure S17. Mass spectrum of the peak (RIB, tr 5.416 min).

Patient 7 (RIB + FUL)

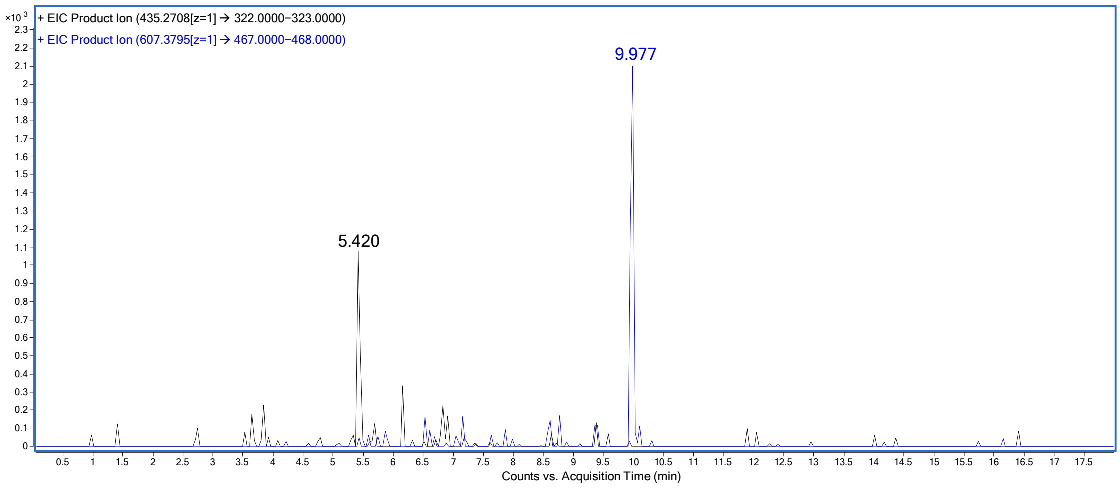

Figure S18. EIC of RIB (transition *m*/*z* 435.3→322.1) and FUL (transition *m*/*z* 607.4→467.2).

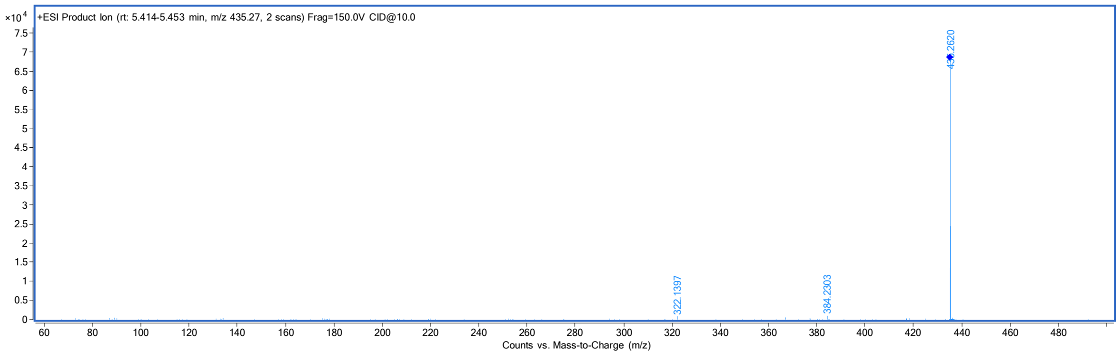

Figure S19. Mass spectrum of peak 1 (RIB, tr 5.420 min).

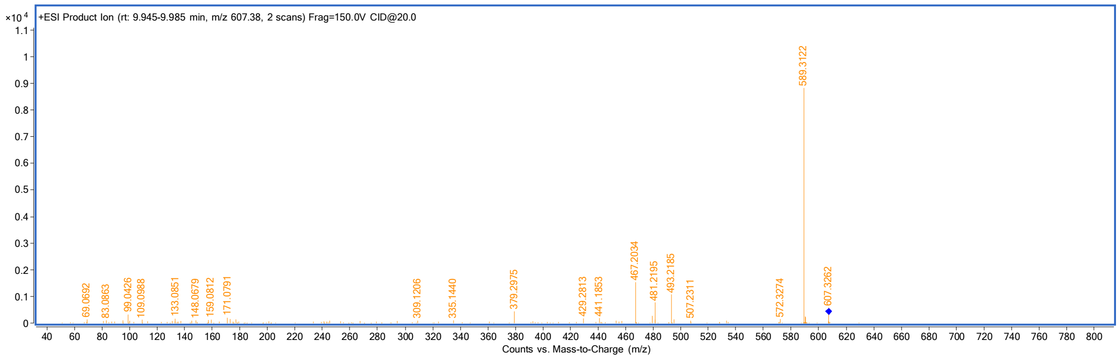

Figure S20. Mass spectrum of peak 2 (FUL, tr 9.977 min).

Patient 8 (PAL + FUL)

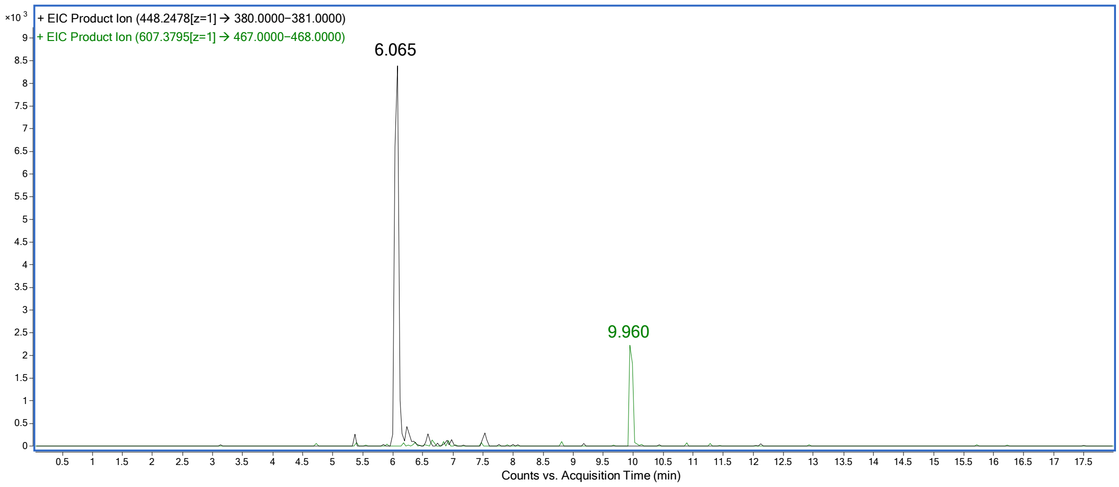

Figure S21. EIC of PAL (transition *m*/*z* 448.3→380.2) and FUL (transition *m*/*z* 607.4→467.2).

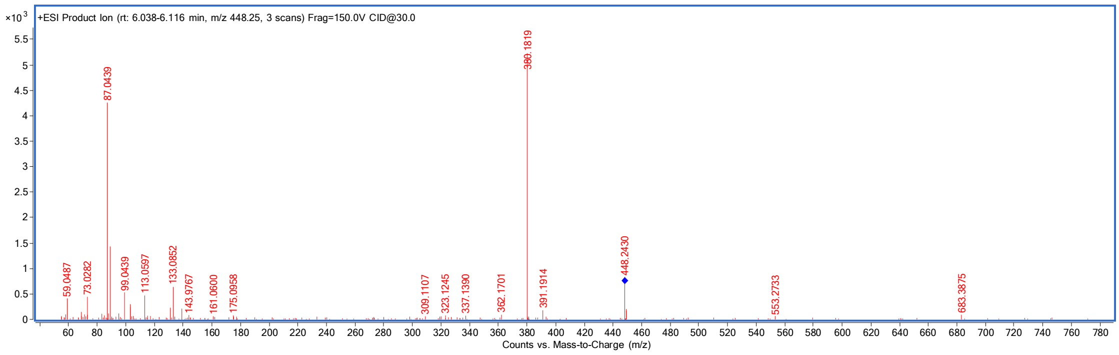

Figure S22. Mass spectrum of peak 1 (PAL, tr 6.065 min).

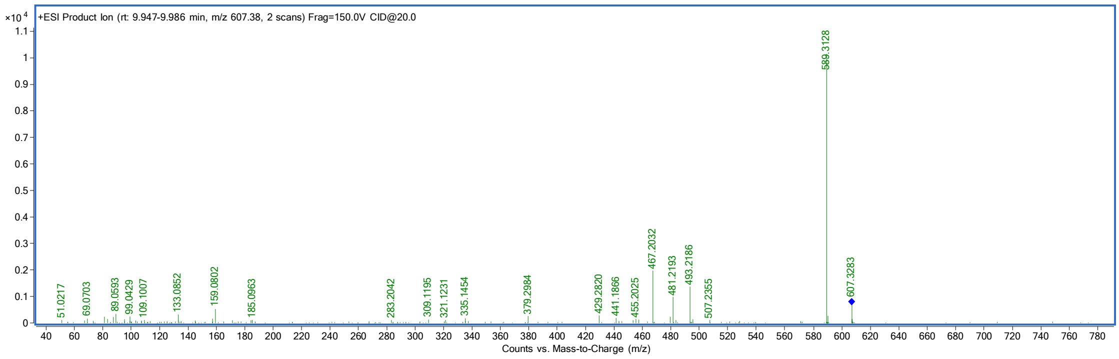

Figure S23. Mass spectrum of peak 2 (FUL, tr 9.960 min).

Patient 9 (PAL + FUL)

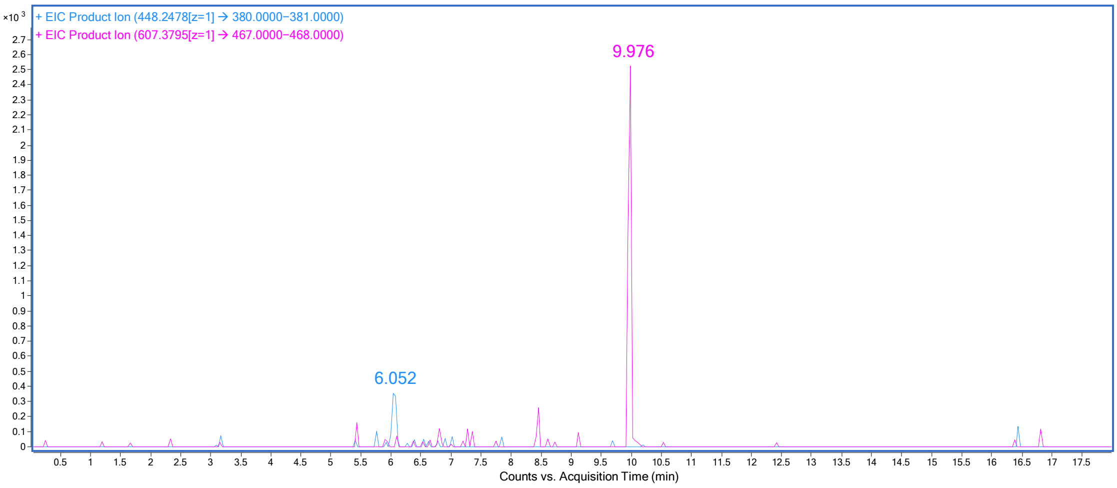

Figure S24. EIC of PAL (transition *m*/*z* 448.3→380.2) and FUL (transition *m*/*z* 607.4→467.2).

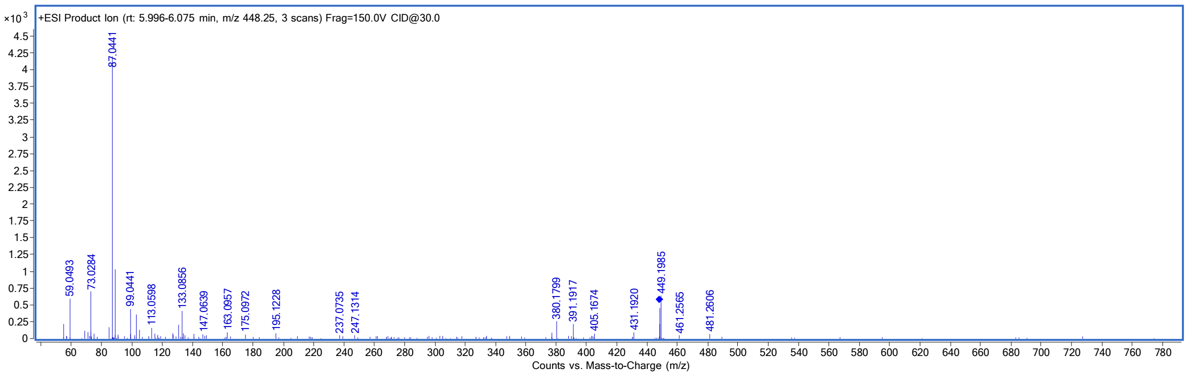

Figure S25. Mass spectrum of peak 1 (PAL, tr 6.052 min).

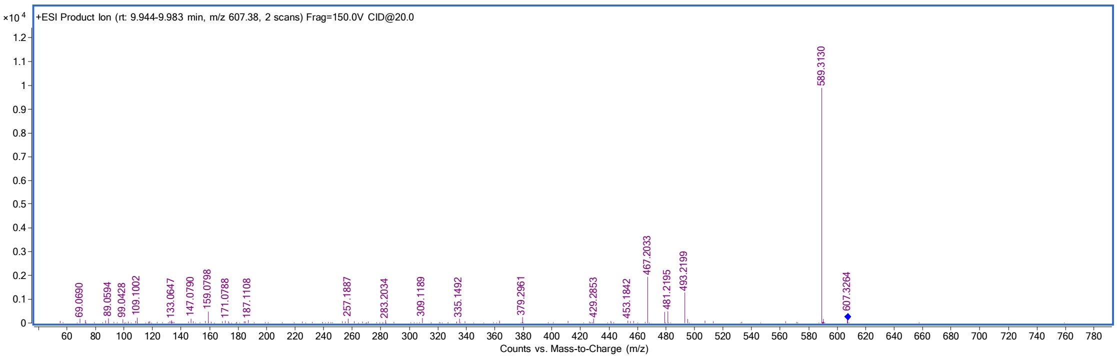

Figure S26. Mass spectrum of peak 2 (FUL, tr 9.976 min).

Patient 10 (PAL + FUL)

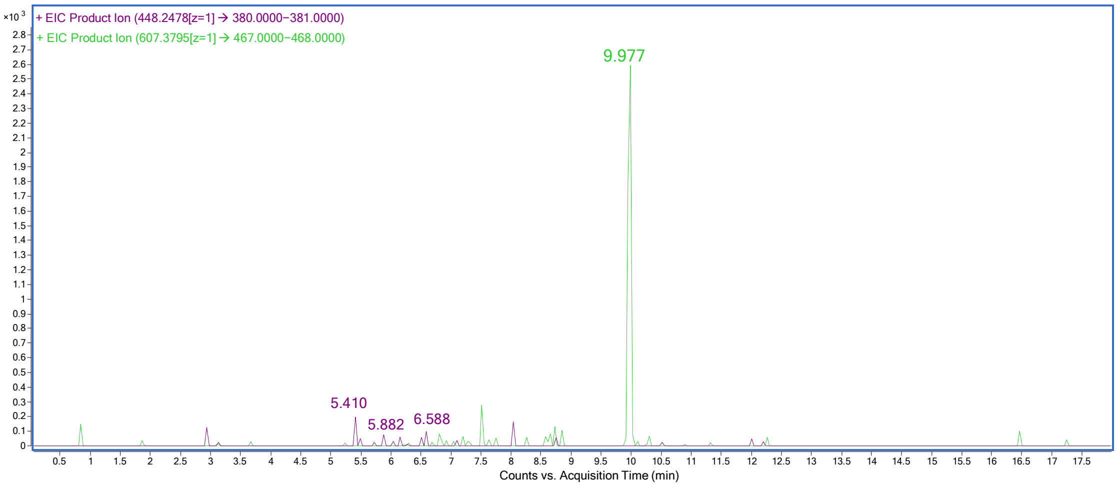

Figure S27. EIC of PAL (transition *m*/*z* 448.3→380.2) and FUL (transition *m*/*z* 607.4→467.2).

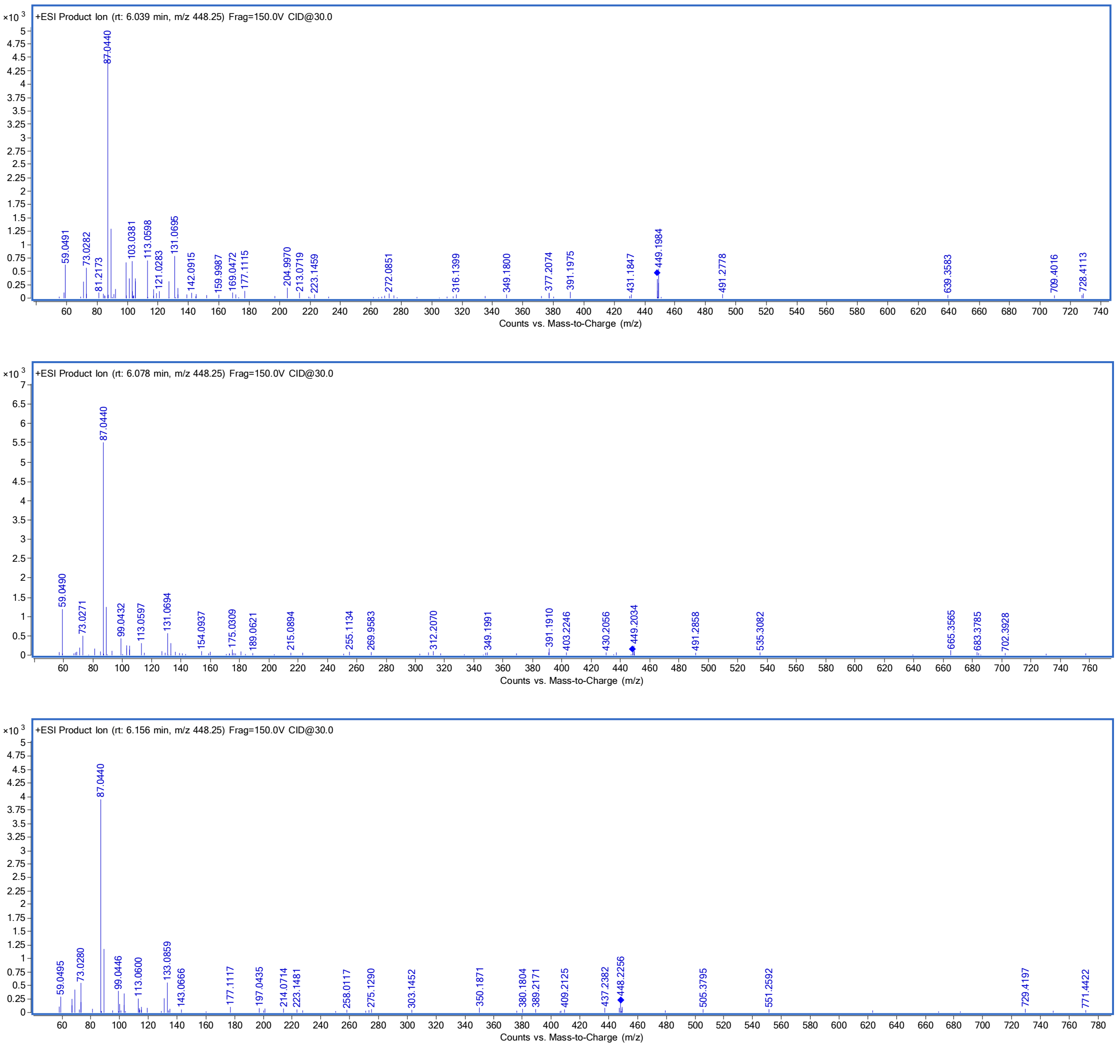

Figure S28. Mass spectra around the retention time of PAL, 6.0 min.

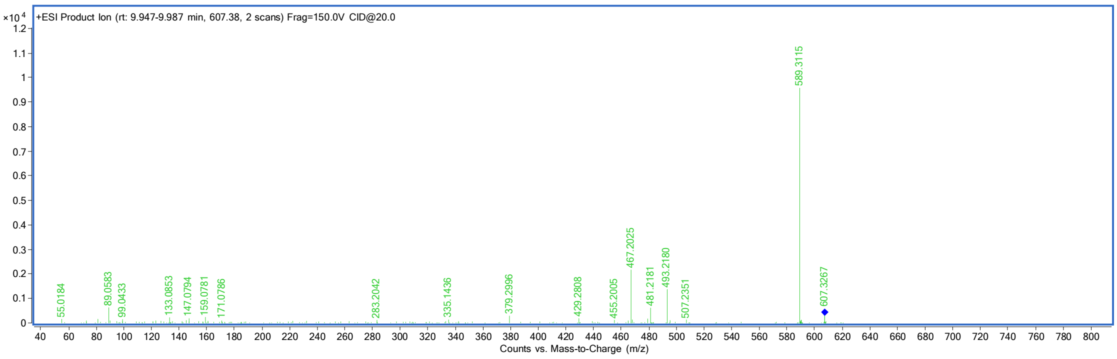

Figure S29. Mass spectrum of the peak (FUL, tr 9.977 min).

Section 2. Results of the pharmacokinetic study:

Table S1. Exact RIB plasma concentrations measured at each timepoint.

**t (h)**	**C_{RIB} (ng/mL)**
0.00	944.84
0.25	1051.75
0.50	1235.13
1.00	1519.81
3.00	1830.10
6.00	1713.58
12.00	1501.88
